# Supplementary material for: The genome sequence of the Orange Sallow moth, Tiliacea citrago Linnaeus, 1758
Source: Wellcome Open Res. 2025 May 20;10:260. [Version 1] doi: 10.12688/wellcomeopenres.24261.1 (PMC12756600; doi:10.12688/wellcomeopenres.24261.1)
Supplement: Peer review history

# Reviewer report: Reviewer response for version 1

**DOI:** 10.21956/wellcomeopenres.26758.r140215

**Published:** 2025-12-31

**Authors:** Yash Gupta

**Affiliations:** 1 Department of Biology, Faculty of Science, Naresuan University, Phitsanulok, Thailand

The manuscript clearly positions the assembly and explains its value as a reference genomic resource for Lepidoptera and biodiversity genomics. The methods are described in a reproducible way (tools, versions, pipeline steps, and key wet-lab procedures are provided), and links to standard operating procedures are included.

 Figure 3 shows a distinct cluster with higher GC (~50–60%) and lower coverage, and the figure legend indicates a phylum assignment including Pseudomonadota. This is a common signature of bacterial contamination or cobionts.If these scaffolds remain in the submitted assembly without clear explanation, downstream users may misinterpret assembly size, gene content, and repeat statistics, and could inadvertently annotate bacterial sequence as moth sequence.

Are sufficient details of methods and materials provided to allow replication by others?

Yes

Is the rationale for creating the dataset(s) clearly described?

Yes

Are the datasets clearly presented in a useable and accessible format?

Yes

Are the protocols appropriate and is the work technically sound?

Yes

Reviewer Expertise:

Genome assembly and Bioinformatics

I confirm that I have read this submission and believe that I have an appropriate level of expertise to confirm that it is of an acceptable scientific standard.

## References

[1] : A reproducible workflow for assembling the mitochondrial genome ofAcheta domesticus (Orthoptera: Gryllidae). *Ecology and Evolution* .2024;14(7) : 10.1002/ece3.11696 10.1002/ece3.11696 PMC1122389338966242

# Reviewer report: Reviewer response for version 1

**DOI:** 10.21956/wellcomeopenres.26758.r124059

**Published:** 2025-06-18

**Authors:** Qing-Song Zhou

**Affiliations:** 1 Institute of Zoology,Chinese Academy of Sciences, Beijing, Beijing, China

This study presents a high-quality genome assembly of the Orange Sallow moth, *Tiliacea citrago*, featuring impressive contiguity and completeness. The methodological rigor and detailed ecological context significantly enhance the value of this genomic resource for Lepidoptera research.

 While the authors report thorough decontamination using the Assembly Screen for Cobionts and Contaminants (ASCC) pipeline, the BlobToolKit GC-coverage distribution in Figure 3 reveals a distinct cluster exhibiting genomic signatures typical of Pseudomonadota (elevated GC content ~50-60% coupled with reduced coverage).

Are sufficient details of methods and materials provided to allow replication by others?

Yes

Is the rationale for creating the dataset(s) clearly described?

Yes

Are the datasets clearly presented in a useable and accessible format?

Yes

Are the protocols appropriate and is the work technically sound?

Yes

Reviewer Expertise:

Entomology genomic diversity

I confirm that I have read this submission and believe that I have an appropriate level of expertise to confirm that it is of an acceptable scientific standard.
